# Aquaporins and their role in plant-microbial systems

**DOI:** 10.18699/vjgb-25-27

**Published:** 2025-04

**Authors:** T.R. Kudriashova, A.A. Kryukov, A.I. Gorenkova, A.P. Yurkov

**Affiliations:** All-Russia Research Institute for Agricultural Microbiology, Pushkin, St. Petersburg, Russia; All-Russia Research Institute for Agricultural Microbiology, Pushkin, St. Petersburg, Russia; All-Russia Research Institute for Agricultural Microbiology, Pushkin, St. Petersburg, Russia; All-Russia Research Institute for Agricultural Microbiology, Pushkin, St. Petersburg, Russia

**Keywords:** aquaporins, AQP, arbuscular mycorrhiza, drought, water transport in plants, symbiosisFor, аквапорины, AQP, арбускулярная микориза, засуха, транспорт воды в растениях, симбиоз

## Abstract

Global losses of agricultural products from water scarcity could be greater than from all other causes combined. Water deficiency in plants can result from insufficient precipitation, elevated air temperatures, and other factors that reduce the water available in the soil. Most terrestrial plants are able to form symbiosis with arbuscular mycorrhizal fungi. Arbuscular mycorrhiza plays a key role in the mineral nutrition of many terrestrial plant species. Water transport in plants is regulated primarily by aquaporins, transmembrane proteins. Aquaporins help plants save water, which is an important component of the plant’s adaptation strategy to water scarcity. Some studies suggest that arbuscular mycorrhizal fungi can decrease the expression of aquaporin genes in plants under drought conditions, which reduces water transport within host plant tissues and conserves available water. On the other hand, there is little scientific evidence of the interaction mechanisms between plants and arbuscular mycorrhizal fungi during aquaporin regulation. In addition, the information in different sources on the aquaporin functions in different plant species may be contradictory. Plant aquaporins are represented by several subfamilies; their number varies for different species. A more comprehensive study of these transporters can enhance our understanding of water transport in plants and assess how arbuscular mycorrhizal fungi can influence it. This review contains data on the history of studies of the structure, localization, phylogeny, and functions of aquaporins. Advancing the study of the symbiotic system functioning may contribute to the development of biofertilizers based on soil microorganisms for agricultural uses in the Russian Federation

## Abstract (ru)

Мировые потери сельскохозяйственной продукции из-за дефицита воды, вероятно, более значительны, чем от других причин, вместе взятых. Причины дефицита воды у растений могут быть связаны с недостатком атмосферных осадков, высокой температурой воздуха и другими факторами, которые могут привести к снижению содержания доступной для растений воды в почве. Большинство наземных растений способно вступать в симбиоз с грибами арбускулярной микоризы. Такой симбиоз выполняет ключевую роль в минеральном питании многих видов наземных растений. Транспорт воды в растениях, ее использование регулируются, в первую очередь, с участием трансмембранных белков – аквапоринов. С помощью аквапоринов растение может «экономить» воду, что является важным элементом стратегии адаптации растения к условиям дефицита воды. По некоторым сведениям, грибы арбускулярной микоризы в условиях засухи способны снижать экспрессию генов аквапоринов растения, тем самым уменьшая транспорт воды внутри тканей растения-хозяина, что приводит к ее «экономии». С другой стороны, в настоящее время в научной литературе информации о механизмах взаимодействия растения и грибов арбускулярной микоризы при регуляции работы аквапоринов недостаточно. Кроме того, имеющиеся в различных источниках сведения о работе аквапоринов у разных видов растений могут противоречить друг другу. Аквапорины в растениях представлены несколькими подсемействами, и их число для разных видов варьирует. Изучение этого семейства транспортеров важно для понимания водного транспорта в растениях и оценки влияния на него со стороны грибов арбускулярной микоризы. В обзоре собраны данные об истории изучения, структуре, локализации, филогении, функциях аквапоринов. Развитие знаний о функционировании симбиотических систем будет способствовать созданию биоудобрений на основе микробной биомассы для использования в сельском хозяйстве Российской Федерации.

## Introduction

Stressful conditions during drought affect plant life in many aspects; under conditions of water deficit, the rate of nutrient uptake from the soil decreases, which has implications for biomass growth and crop yields (Ahanger, Agarwal, 2017). Proteins from the Aquaporin family (AQP) are involved in the transport of water in plants. This family is part of a larger major intrinsic proteins (MIP) family (Nielsen et al., 2002; Zhou Y., MacKinnon, 2003). This family received its name after the first water transporter was found in the lens fibers of mammals (including humans), which was later named Aquaporin 0. Aquaporins are represented by integral membrane proteins forming transmembrane pores in cells. During a genomic AQP family study across various plants (24 species including algae, mosses, lycophytes, dicotyledons, and monocotyledons), the aquaporins were divided into eight subfamilies, evolving from large intrinsic proteins (LIPs), found in diatom algae, to tonoplast intrinsic proteins (TIPs) (Hussain et al., 2020). Five out of the eight MIP subfamilies are found in seed plants (including monocotyledons and dicotyledons): plasma membrane intrinsic proteins (PIPs), tonoplast intrinsic proteins (TIPs), nodulin 26-like intrinsic proteins (NIPs), small basic intrinsic proteins (SIPs), and X intrinsic proteins (XIPs) (Danielson, Johanson, 2008). Hybrid intrinsic proteins (HIPs) and GLpF-like intrinsic proteins (GIPs) are found only in mosses (Abascal et al., 2014; Singh et al., 2020).

Arbuscular mycorrhizal fungi (AMF) enhance nutrient uptake, particularly that of phosphorus, in host plants while also regulating their water balance and transport (Schachtman et al., 1998; Huey et al., 2020). At the same time, mycorrhization changes the regulation of AQP genes in plants. In arbuscular mycorrhizal (AM) symbiosis, the root cortical cells form a periarbuscular membrane that surrounds each arbuscule, creating a separation between the fungus and the plant cytoplasm. This process establishes a plant-fungus relationship, helping the host plant obtain water and nutrients, and improving drought tolerance (Kakouridis et al., 2022). In synergy with other microorganisms, an arbuscular mycorrhiza has the potential to supersede classical chemical fertilizers that have adverse effects on ecosystems and reduce land degradation caused by drought in particular (Kuila, Ghosh, 2022; Seka et al., 2022). It is suggested that reducing aquaporin expression during water deficiency may be a way to minimize water loss (Quiroga et al., 2017). The review is aimed to study the impact of AM on plant water exchange and the AQP family’s role in increasing plant drought tolerance

## Background. Aquaporin structure

Water transport in the plant is mediated by three pathways: apoplastic, symplastic, and transmembrane transport. The latter involves aquaporins present on biological membranes and forming channels (Singh et al., 2020). Such channels ensure the movement of water in two directions (Chrispeels, Agre, 1994).

Aquaporins were discovered in the 1980s when researchers studied the mechanisms of water transport across cell membranes. Peter Agre began studying red blood cell proteins at Johns Hopkins University in 1988. He discovered a protein that was bound to antibodies targeting glycophorin. This protein was called CHIP28 (channel-forming integral protein) (Agre et al., 1993).

Although the basic concept of water movement across biological membranes had already been established in the early 1950s, G.M. Preston discovered the first CHIP28 water channel in human erythrocytes only in 1992 (Preston et al., 1992); it was subsequently named Aquaporin-1 (AQP-1). Later, a similar protein was identified in Escherichia coli and named aquaglyceroporin (GLP-F).

In 1992, P. Agre and colleagues cloned the AQP1 gene to determine its structure. AQP1 is a transmembrane protein with four transmembrane domains that form a narrow channel through which water molecules can move (Agre et al., 1993). The first identified plant aquaporin was AtTIP1;1, a transmembrane protein found on the vacuolar membrane of Arabidopsis thaliana (Maurel et al., 1993). By 1999, MIPs included 150 representatives that had been identified on cell membranes of various organisms, from bacteria to humans (Lagrée et al., 1999). To this date, more than 7,541 MIP homologs have been discovered in 484 eukaryotic species (Irisarri et al., 2024). MIP family proteins are confirmed to localize on the cell membranes of all living organisms. Plasma and inner membranes as well as viral envelopes are key localization sites for MIPs. The Nobel Prize awarded to Peter Agre and Roderick MacKinnon for the discovery of AQPs in 2003 (discovery of the 3D molecular structure of the bacterial potassium channel and the explanation of the nature of their selectivity) brought AQPs into the spotlight (Knepper, Nielsen, 2004). This discovery demonstrated how water can rapidly and efficiently pass through cell membranes despite their hydrophobic nature.

The function of aquaporins in the water uptake and transport in mycorrhizal plants has been investigated since 1997. Initial studies revealed the expression of aquaporin genes of the TIP subfamily in Medicago (Medicago truncatula) and Petroselinum (Petroselinum crispum) inoculated with AMF. The first analysis of transport properties was performed for MtAQP1 (Krajinski et al., 2000). RiAQP1, the first AMF AQP, is believed to have been found in Rhizophagus irregularis in 2009. RiAQP1 was distinctively expressed during cold and drought stress in the roots of the host plant (Aroca et al., 2009). To the present day, Russian and foreign scientists continue the vigorous studies of the aquaporin functions in various tissues and organs in genetics, biotechnology, medicine, and agriculture.

All members of the aquaporin family in plants have a similar structure. An aquaporin is a tetramer composed of monomers, each with six transmembrane domains (1–6) and five connecting loops (A–E) localized on the intra- (B, D) or extracytosolic (A, C, E) side of the membrane. Loops A and D have an asparagine-proline-alanine sequence (the NPA motif) and form hydrophobic α-helixes. Each NPA sequence is oriented toward the center of the AQP pore. The sequences contribute to the contraction of the central pore and, in combination with the dipole moment of two α-helixes enveloping the membrane, prevent proton (H+) permeation (see the Figure).

**Fig. 1.**
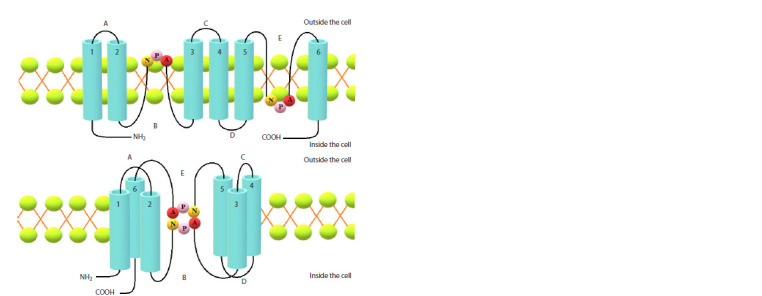
Aquaporin protein structure according to (Kapilan et al., 2018), with revisions.

Both aquaporin terminal ends (N and C) are oriented to the cytoplasmic side of the membrane and help carry out the specific regulation of the aquaporin activity. In addition, four conserved sequences form a typical aromatic arginine constriction near the extracytosolic pore mouth that functions as the main selectivity filter (Hussain et al., 2020). Aquaporin activity is regulated by post-translational modifications (phosphorylation and methylation), pH, Ca2+, and interactions between aquaporin monomers, whereas the AQP substrate specificity is determined by its structure (Wang Y. et al., 2020).

## AQP phylogeny and subfamilies

MIP family genes, including AQPs, are characterized by a larger number of isoforms in plants compared to animals, as plant cells are more compartmentalized. AQPs in plants vary by species, subcellular localization, solute water-transmitting capabilities, and function (Chaumont, Tyerman, 2014; Afzal et al., 2016).

Based on their structural features and functional diversity, AQPs can be roughly divided into four subfamilies. The first subfamily functions as a water-permeable channel, the so-called Classical AQP (C-AQP). The second subfamily is Aquaglyceroporin (AQGP); it is responsible for glycerol transport. The third subfamily includes proteins with highly degraded NPA motifs, the functions of which are yet to be identified. This subfamily has been termed superaquaporins or subcellular aquaporins (SAQPs). SAQPs may be involved in the transmembrane transport of ammonia and some other molecules. This subfamily is AQP-8 (Jia, Liu, 2020).

In model plants, the aquaporin family is represented by different numbers of transporters: Avena sativa has 45 AQP genes, A. thaliana – 38, Solanum lycopersicum – 47, Physcomitrella patens – 35, Gossypium hirsutum – 74, Zea mays – 41, Oryza sativa – 33, Populus trichocarpa – 54, and Glycine max – 66. Rapeseed (Brassica napus) contains the highest number of AQPs, 121; including, PIP – 43, TIP – 35, NIP – 32, and SIP – 11 (Hussain et al., 2020; Zhou X. et al., 2024).

M. truncatula, a well-known model plant, contains 46 identified putative loci encoding genes from five aquaporin subfamilies, including 10 PIPs, 12 TIPs, 18 NIPs, 4 SIPs, and 2 XIPs. The first four subfamilies were further divided into 2 (PIP1-PIP2), 5 (TIP1-TIP5), 7 (NIP1-NIP7), and 2 (SIP1-SIP2) corresponding subgroups; the XIP subfamily has one subgroup with only two representatives (Min et al., 2019). Based on homology, PIPs are divided into two subgroups, PIP1 and PIP2. The differences between these two subgroups lie in the water-transmitting capabilities of these proteins; PIP1 has longer N-terminal but shorter C-terminal ends compared to PIP2 (C-terminal ends have an additional 4–10 amino acid region in the first extracytosolic loop). PIP1 and PIP2 have five and eight isoforms, respectively. These two subgroups interact through hetero-oligomerization, in which two PIP2 monomers form heterotetramers with two PIP1 monomers (Wang Y. et al., 2020).

TIPs have more isoforms than PIPs and are divided into five protein subgroups. For example, in P. trichocarpa, 17 TIPs are present among 55 MIP sequences (Kapilan et al., 2018). For Cicer arietinum L., all TIPs are phylogenetically divided into 14 subgroups. The phylogenetic tree shows that out of 21 branches, only four are interspecific, and the rest are intraspecific. Their functionality for plant species is expanding (Hussain et al., 2020).

NIPs in plants also have numerous isoforms and can be divided into five subgroups. NIP subgroups are found in all higher plants, although NIP3 is found mainly in monocotyledons (Lu et al., 2018). In particular, eleven NIPs have been discovered in P. trichocarpa (Gupta, Sankararamakrishnan, 2009). NIPs were found in G. max, C. arietinum, and Phaseolus vulgaris as a result of symbiosis with nitrogenfixing bacteria. NIP sequences vary significantly both within and between species (Hussain et al., 2020). Most NIPs bear similarity to the nodulin 26 protein, which is expressed in the symbiosome membrane under conditions of inoculation with rhizobacteria (Kapilan et al., 2018).

SIPs are small, just like TIPs. The main reason for their compact size is a very short cytosolic N-terminal region, as compared to other plant AQPs. Based on the NPA motif, SIP1 is divided into SIP1;1 and SIP1;2. Different SIP isoforms have different water-transmitting capabilities for solutes (Kapilan et al., 2018).

The first identification of the relatively recently discovered XIP subfamily was carried out for G. hirsutum in 2010 (Park et al., 2010). Nineteen representatives of XIP are known to date, including five XIPs in P. trichocarpa. The remaining ten XIP representatives have been found in other dicotyledonous plants, three – in mosses, and one – in protozoa. XIP homologs have not been discovered in monocotyledonous plants. Expression analysis in P. trichocarpa demonstrates that XIPs in poplar do not manifest an abundance of tissuespecific transcripts (Gupta, Sankararamakrishnan, 2009; Kapilan et al., 2018).

The high number of AQP isoforms within subfamilies may contribute to enhanced transporter functions and plant adaptation to changing conditions. M. truncatula AQP molecular structure analysis revealed that among aquaporins, seven genes (15.2 %) exhibit tandem duplication, and ten genes (21.7 %) exhibit segmented duplication (Min et al., 2019). In other words, the presence of many isoforms and distinct subgroups of different aquaporin subfamilies underscores their substantial importance in living organisms.

## Aquaporin localization and functions in plants

Maurel C. et al. (2015) showed that in addition to water, some members of the MIP superfamily, including aquaporins, can also transport glycerol, carbon dioxide, urea, ammonia, hydrogen peroxide, boron, silicon, arsenic, antimonite, lactic acid, and oxygen across membranes. Some AQPs are capable of transmitting univalent cations (Byrt et al, 2017). AQPs may be involved in the signaling of such hormones as auxin, gibberellins, ethylene, and abscisic acid (ABA) in plants (Wang C. et al., 2016). The aquaporin subfamilies localization and functions are listed in the Table.

**Table 1.**
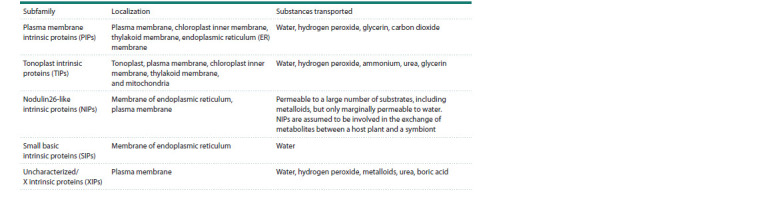
Cellular localization and plant aquaporin functions* According to (Ishikawa et al., 2005; Kruse et al., 2006; Ma et al., 2006; Maurel et al., 2015; Pommerrenig et al., 2015; Lopez et al., 2016; Noronha et al., 2016; Wang C. et al., 2016; Byrt et al., 2017; Kapilan et al., 2018; Zhou X. et al., 2024).

Embryophytes are known to have evolved with a remarkably high degree of subcellular compartmentalization. In this respect, in contrast to animals, plant aquaporins show a wider range of subcellular localizations (plasma membrane, tonoplast, chloroplast, endoplasmic reticulum, Golgi apparatus, and mitochondria), with AQPs capable of simultaneous localization in different cells and on different membranes. Representatives of the same subfamily may have different subcellular localizations (Zhou X. et al., 2024).

PIPs are mainly localized on the plasma membrane, typically in tissues characterized by high water transport; for example, in conducting tissues (Yaneff et al., 2014). PIP transporters are also found in A. sativa, on the endoplasmic reticulum (Zhou X. et al., 2024). PIP1 subgroup transporters typically localize at the plasma membrane (Kaldenhoff, Fischer, 2006) and have low water-transmitting capabilities (Kapilan et al., 2018). Some PIP1 proteins are unable to act independently, and they must form heterotetramers with PIP2 monomers to be able to increase water-transmitting capabilities (Schuurmans et al., 2003). In tobacco (Nicotiana tabacum) plants, decreased expression of NtAQP1, a PIP1 family member, caused decreased water transport in roots and reduced plant resistance to water stress. In peas (Lathyrus oleraceus), PIP1 has been found to play an important role in seed water supply (Kaldenhoff, Fischer, 2006).

Some researchers have noted an additional function of the PIP1 subgroups in plant cells – the ability to transport CO2 (Kapilan et al., 2018). Increased OsPIP2;7 expression in rice improves its survival under low-temperature stress and affects the expression levels of other AQP genes (Zhou X. et al., 2024).

The PIP1 and PIP2 subgroups are believed to be localized in almost all plant parts, including roots and leaves (Maurel et al., 2015). The PIP2 subfamily aquaporins are more efficient as water channels than PIP1 group members; various PIP2 isoforms are considered to be major transporters of water across the cell membrane (Kaldenhoff, Fischer, 2006). The expression patterns of the AQP gene in A. thaliana differed significantly under drought stress (e. g., decreased AtPIP2;1 and AtPIP2;2 expressions under stress). The expression analysis for OsPIP1 in rice under drought stress showed that the expression of these two genes was elevated, whereas the expression of all OsPIP2 genes was reduced. These results indicate that the expression of AQP genes in plants under drought is regulated by a complex signaling network, and the mechanism of AQP gene regulation in plant drought tolerance requires further investigation (Zhou X. et al., 2024).

TIPs are mainly localized on vacuolar membranes (Johnson et al., 1990). The five TIP aquaporin subgroups (in Arabidopsis, maize, and rice) are mainly located in tonoplasts, but some TIP isoforms have also been found at the plasma cell membrane. In Avena, seven TIP representatives are of cytoplasmic origin, and five TIPs are localized in the tonoplast (Zhou X. et al., 2024). Because of the high aquaporin content in the tonoplast, the water transmission of the tonoplast is believed to be much higher than that of the plasma membrane. This contributes to the turgor pressure within the cell (Luo et al., 2022). In addition to functions of aquaporins in water transfer, TIPs are known to participate in the transport of urea, glycerol, and ammonia and are also involved in the plant response to abiotic stress (Loque et al., 2005). Several TIP isoforms show important roles in plant response to drought. In 2020, A. Lopez-Zaplana et al. observed the presence of a TIP on mitochondrial membranes (Lopez-Zaplana et al., 2020). TIP1 and TIP2 isoforms are believed to be expressed in plant vegetative tissues, TIP3 isoforms are mainly expressed in seeds, and TIP5 isoforms are associated with pollen grains (Hussain et al., 2020). TIP is a highly specific subfamily and can fulfill a variety of functions in different plant species.

Nodulin 26-like intrinsic proteins were initially identified in the peribacteroid membrane in G. max nodule. GmNOD26 was the first to be described (Fortin et al., 1987). NIPs are thought to be involved in metabolite exchange between a host plant and a microsymbiont (Kruse et al., 2006). Although the GmNOD26 gene product localized exclusively in the peribacteroid membrane of nitrogen-fixing symbiotic legume nodule, NOD26-like proteins (NIPs), forming the third subfamily, can also occur in plant species other than legumes in which they localize in the plasma membrane (Ma et al., 2006) or the ER membrane (Mizutani et al., 2006).

Therefore, NIPs are widespread not only in Leguminosae (forming a rhizobia-legume symbiosis), indicating that they can function in the absence of symbiotic relationships (Wayne, Tazawa, 2010). For example, X. Zhou et al. showed that in A. sativa the expression of the NIP subfamily 6Ag0000836.1 gene was significantly upregulated under various abiotic stresses. This gene is suggested to be a marker of response to abiotic stress (Zhou X. et al., 2024). Although NOD26 and other NIPs have lower water-transmitting capabilities compared to other aquaporins, they also have a transport function to carry glycerol (Kaldenhoff, Fischer, 2006). This may indicate that the common ancestor of different AQP groups in plants did not have a glycerol transport function (Zhang et al., 2020).

The subcellular localization of most NIP transporters is not entirely clear. NIPs can localize on the membrane of the endoplasmic reticulum (Lopez et al., 2016). In A. thaliana, NIP5;1 is localized in the plasma membrane, while NIP2;1 is localized on the ER membrane (Zhou X. et al., 2024). In addition, NIPs can transport ammonia, lactic acid, boron, and silicon (Danielson, Johanson, 2008). NIPs are also metalloid transporters (Pommerrenig et al., 2015); NIPs not only facilitate metalloid diffusion from the soil but also play a key role in their transport in the plant. Therefore, we can hypothesize that the NIP family promotes the uptake and translocation of metalloids, thus regulating their amount.

The group of small SIPs is localized on the ER membrane (Ishikawa et al., 2005). The SIP subfamily in plants is inadequately studied in terms of their structure and functionality (Hussain et al., 2020). SIP structure differs from other AQP subfamilies since their cytosolic N-terminal region is relatively shorter (Kapilan et al., 2018). Due to their shortened NPA motif, SIPs can also transport different molecules, not just water. Currently, there is no consensus on the role of SIPs in transport in plants. In a study of A. thaliana, only the aquaporins SIP1.1 and SIP1.2 (out of three subfamily aquaporins) demonstrated minor water-transmitting capabilities, while SIP2.1 did not exhibit this function (Ishikawa et al., 2005). SIP1 proteins transport water across the ER membrane, while the SIP2 protein acts as an endoplasmic reticulum channel for small molecules or ions (Hussain et al., 2020). To date, A. sativa has been found to have two SIP genes (4Dg0000047.3 and 5Ag0000631.1) that show expression in tissues of the aboveground plant organs (Zhou X. et al., 2024).

Members of the XIP subfamily are found in protozoa, fungi, and plants, but their functions are to be studied further (Kapilan et al., 2018). In tobacco plants, the XIP family gene products, NtXIP1;1α and NtXIP1;1β, are localized at the plasma membrane. NtXIP1;1, however, showed expression in all plant tissues. This subfamily is found to be absent in plants such as Arabidopsis, maize, and rice. The sequences of the XIP subfamily are shorter compared to those of other MIP subfamilies. However, their structure remains highly conserved and similar to other subfamilies (Wang C. et al., 2016). XIPs exhibit contrasting transport functions in different plant species (Lopez-Garcia et al., 2018). For example, in grapevine, VvXIP1 is active in osmotic regulation in addition to H2O2 transport and metalloid concentration regulation (Noronha et al., 2016). Heterologous expression of solanaceous XIP family genes in Xenopus laevis oocytes and various yeast strains of Saccharomyces cerevisiae showed that these isoforms contribute to the transport of large molecules such as glycerol, urea, and boric acid. Water-transmitting capabilities, however, were not identified. This indicates that XIPs are involved in the transport of non-charged molecules across the plasma membrane of cells in certain plant tissues (Kapilan et al., 2018).

## Aquaporins in plant-microbial systems

Microbial-plant relationships form the foundation of life on Earth. These interactions can be specific and evolutionarily reinforced or non-specific, temporary, and random. It is known that embryophytes can enter into symbiotic relationships with microorganisms. These relationships can be of different types: mutualism, commensalism, amensalism, parasitism, or neutralism (Yatsenko-Stepanova et al., 2014). Symbiotic relationships depend on the conditions in which they exist. In other words, the same host-symbiont combination can be mutually beneficial in one case but parasitic in another (Chiu, Paszkowski, 2019). For A. thaliana, the Colletotrichum toefieldiae fungus was found to be beneficial only under phosphorus-deficient conditions, while in other situations it acted as a parasite (Hiruma et al., 2016).

Plants face constant threats from pathogens such as viruses, bacteria, and fungi. These attacks result in various diseases affecting crucial crops, leading to significant food losses (Savary et al., 2019). Growing evidence suggests that AQPs play a role in plant defense against pathogens by modulating plant immunity and resistance to invasive diseases (Li et al., 2020). Bacterial pathogen-induced AtPIP1; 4 transports water from the apoplast to the cytoplasm to activate systemic resistance and immune responses in A. thaliana (Tian et al., 2016). Plants can close their stomas to conserve moisture after perceiving molecular patterns associated with the pathogen to limit the invasion. The stress hormone ABA is found to be involved in the regulation of stoma closures. AtPIP1;2 has been shown to facilitate water transport across the plasma membrane, causing ABA- and pathogen-induced closure of stomas in A. thaliana (Exposito- Rodrigues et al., 2017).

During the initial stages of infection, the fungal pathogens regulate their development to send the special infectious hyphae into the host organism to obtain nutrients. In Fusarium graminearum, the FgAQP1 protein localized in the nuclear envelope in conidia is important for hyphal growth, development, and secondary metabolism. Deletion in FgAQP1 affects gene expression, which reduces plant infection efficiency, suggesting FgAQP1 may play a key role in the interaction between F. graminearum and the host (Ding et al., 2018).

At the same time, bacteria are known to stimulate plant growth under both favorable and stressful conditions (Pseudomonas mandelii, Rhizobium leguminosarum bv. viciae, etc). E. Martynenko et al. showed the relationship between AQP and the formation of apoplastic barriers in the plant-microbial system “Pisum sativum + P. mandelii”. P. mandelii increases aquaporin activity, which compensates for a possible decrease in water-transmitting capabilities in pea roots (Martynenko et al., 2023).

Compared to pathogens, AMF in root cortex do not disrupt host plant cell integrity (Mosse et al., 1981; Spatafora et al., 2016). Nutrient exchange and water transport between symbionts occur in arbuscules (Zhang et al., 2019). In addition, AMF colonization of plants promotes the smooth closure of plant stomas during drought. AM symbiosis improves the transporting capabilities of stomas and leaf transpiration to adapt to arid environmental conditions (Ni et al., 2024). Acting as “extended plant roots”, AMF improve photosynthetic efficiency and osmoregulation, and enhance plant antioxidant metabolism (Evelin et al., 2019). AQPs can be divergently expressed in response to mycorrhization (Asadollahi et al., 2023). When a plant is under stress, AMF could affect AQP expression at transcriptional, translational, and posttranscriptional (AQP phosphorylation, multimerization, cycling, and internalization) levels, which contributes to the active regulation of AQP expression and protein abundance, thereby improving the efficiency of transport of H2O, CO2, glycerol, NH3, etc. (Kakouridis et al., 2022).

In the plant-microbial system (PMS) “Triticum aestivum + R. irregularis (formerly known as Glomus intraradices Schenck & Smith)”, it has been shown that AMF colonization of plants activates genes involved in the phenylpropanoid biosynthesis pathway and transcription factors that play a vital role in plant defense against biotic and abiotic stresses (Mashini et al., 2022).

It is suggested that AM colonization is associated with modifications of membrane transporters, especially aquaporin proteins. For example, differential expression of genes due to water deficit was analyzed in the “T. aestivum + Funneliformis mosseae” PMS: TaPIP1-6 and TaPIP1-8 from the PIP1 subfamily; PIP2 – TaPIP2-2C1, TaPIP2-2C3, TaPIP2-3C1, TaPIP2-7, TaPIP2-22; NIP3 – only TaNIP3-1; TIP4 – TaTIP2-5, TaTIP4-1, TaTIP4-2, TaTIP4-6. The AQP genes with downregulated expression belonged to the PIP1, PIP2, TIP2, TIP4, and NIP subfamilies. Gene products were localized at the plasma membrane or tonoplast. In contrast to other AQPs, in addition to its function as a water transporter, TaNIP3-1 also showed activity in the transmembrane transport of arsenite and boronic acid salts. In the “T. aestivum + F. mosseae” PMS, water deficit did not affect SIP expression. In wheat, 25 out of the 96 known aquaporin genes changed their expression when inoculated with an AM fungus. At the same time, only four genes showed increased expression: TaNIP1-10, TaNIP3-3, TaNIP3-4, and TaTIP1-5. Half of the analyzed AQPs with reduced gene expression in wheat were localized at the plasma membrane, while the rest were localized at the tonoplast (TIP1, TIP2, TIP4, PIP1, PIP2, NIP2, SIP2, and NIP3). It is of interest that the expression of TaPIP2-2C3, TaPIP2-2C1, TaTIP4-6, TaPIP1-6, and TaPIP2-3C1 was suppressed in mycorrhized plants under water deficit, while the expression of TaNIP1-10, TaNIP3-3, TaNIP3-4, TaNIP1-5, and TaPIP2-7 was elevated under the same conditions (Asadollahi et al., 2023).

In maize, AM symbiosis is known to suppress several aquaporins, including ZmPIP1-1, ZmPIP1-3, ZmPIP1-4, ZmPIP1-6, ZmPIP2-2, ZmPIP2-4, ZmTIP1-1, and ZmTIP2- 3, but enhances the expression of TIP4-1. At the same time, the drought-tolerant maize variety showed different results. Only three of the AQP genes under study (ZmPIP1;6, ZmPIP2;2, and ZmTIP4;1) changed expression upon symbiosis with AMF. The results of the experiment with a drought-tolerant maize strain are consistent with the hypothesis that reducing aquaporin gene expression under water deficit may be a way to minimize water loss (Quiroga et al., 2017).

Expression of GiAQP1, RiAQPF1, and RiAQPF2 was also evaluated in “Daucus carota + R. irregularis” PMS under drought conditions. Only RiAQPF2 was expressed differentially (Keller-Pearson et al., 2023).

Joint colonization of maize with R. irregularis and Exophiala pisciphila (dark septate endophyte, DSE) resulted in high water transmission through stomas and downregulation of the ZmPIP1;1, ZmPIP1;2, ZmPIP2;1, ZmPIP2;5, and ZmPIP2;6 gene expression compared with reference plants without microbial infection and with individual fungus colonization. The GintAQPF1 and GintAQPF2 expression in R. irregularis has been shown to change significantly under drought stress conditions. The competitive relationship between AMF and DSE in mycorrhization during the experiment is also worth noting. On the other hand, AMF and DSE are also known to form synergistic relationships to regulate membrane electrolyte transport, oxidative stress, photosynthesis, and aquaporin expression; such a relationship has been studied in maize seedlings (Gong et al., 2023).

D. Wang et al. showed that the ZmTIP2;3 gene expression in the “Z. mays + R. irregularis (previously attributed to Glomus intraradices)” PMS was significantly upregulated under drought conditions through AMF symbiosis. ZmTIP2;3 is an aquaporin with six transmembrane domains and two highly conserved NPA motifs. Its promoter region contains many cis-acting elements associated with the induction of AM symbiosis. In the experiment, ZMTIP2;3 gene mutation resulted in decreased biomass, colonization rate, photosynthesis, proline, and expression levels of several drought-related genes (LEA3, P5CS4, and NECD1) compared with the wild post-AMF-inoculation type under drought conditions. This suggestes that ZmTIP2;3 enhances drought tolerance in maize through symbiosis with the AM fungus (Wang D. et al., 2024).

## Conclusion

The discovery of aquaporins was a major event in biology and medicine. The main function of aquaporins is the regulation of transmembrane water transport both between and within cells (Maloy, Hughes, 2013). Aquaporin isoforms can vary greatly depending on the organism type and the living conditions. Depending on the environmental conditions, the aquaporin activity may change, as well as their function in regulating water transport. Such strategic patterns of changes in aquaporin gene expression and functional diversity are the basis for adaptation to environmental changes, including stress-induced ones (Jia, Liu, 2020). A significant number of host plant aquaporins are regulated in AMF symbiosis. The regulation of their genes may depend on the magnitude of drought stress. Some of these aquaporins can transport other molecules of crucial importance, in addition to water. The results of studies carried out under a wide variety of conditions confirm that mycorrhized plants grow and develop better than plants without mycorrhization. At the same time, mycorrhized plants are more efficient in the preservation and transfer of water between tissues, nitrogen compound mobilization efficiency is increased, as well as glycerol accumulation, synthesis of signaling molecules, and accumulation of metals that play a role in resistance to abiotic stresses.

The continued exploration of this topic will enhance our understanding of the specific roles played by aquaporin isoforms in response to arbuscular mycorrhizal symbiosis. This research will help us determine how this symbiosis influences plant adaptation mechanisms under stress conditions. By monitoring the transcriptional responses of aquaporin genes to various environmental factors, we can deepen our knowledge and contribute to the development of biotechnological programs aimed at improving crop resilience.

## Conflict of interest

The authors declare no conflict of interest.
